# Child Growth According to Maternal and Child HIV Status in Zimbabwe

**DOI:** 10.1097/INF.0000000000001574

**Published:** 2017-08-18

**Authors:** Adetayo O. Omoni, Robert Ntozini, Ceri Evans, Andrew J. Prendergast, Lawrence H. Moulton, Parul S. Christian, Jean H. Humphrey

**Affiliations:** From the * Department of International Health, Johns Hopkins Bloomberg School of Public Health, Baltimore, Maryland; † Zvitambo Institute for Maternal and Child Health Research, Harare, Zimbabwe; and ‡ Blizard Institute, Queen Mary University of London, London, United Kingdom.

**Keywords:** HIV, children, growth, stunting, wasting, Zimbabwe

## Abstract

**Background:**

Growth failure is common among HIV-infected infants, but there are limited data on the effects of HIV exposure or timing of HIV acquisition on growth.

**Methods:**

Fourteen thousand one hundred ten infants were enrolled in the Zimbabwe Vitamin A for Mothers and Babies trial in Zimbabwe before the availability of antiretroviral therapy or co-trimoxazole. Anthropometric measurements were taken from birth through 12–24 months of age. Growth outcomes were compared between 5 groups of children: HIV-infected in utero (IU), intrapartum (IP) or postnatally (PN); HIV-exposed uninfected (HEU); and HIV unexposed.

**Results:**

Growth failure was common across all groups of children. Compared with HIV-unexposed children, IU-, IP- and PN-infected children had significantly lower length-for-age and weight-for-length Z scores throughout the first 2 years of life. At 12 months, odds ratios for stunting were higher in IU [6.25, 95% confidence interval (CI): 4.20–9.31] and IP infants (4.76, 95% CI: 3.58–6.33) than in PN infants (1.70, 95% CI: 1.16–2.47). Compared with HIV-unexposed infants, HEU infants at 12 months had odds ratios for stunting of 1.23 (95% CI: 1.08–1.39) and wasting of 1.56 (95% CI: 1.22–2.00).

**Conclusions:**

HIV-infected infants had very high rates of growth failure during the first 2 years of life, particularly if IU or IP infected, highlighting the importance of early infant diagnosis and antiretroviral therapy. HEU infants had poorer growth than HIV-unexposed infants in the first 12 months of life.

In 2013, an estimated 1.4 million pregnant women were living with HIV worldwide, the majority in sub-Saharan Africa.^1^ In the absence of antiretroviral therapy (ART) for prevention of mother-to-child transmission (PMTCT), up to 40% of infants born to HIV-infected women acquire the infection^2^ and, without treatment, around half will die before their second birthday.^3^ HIV is transmissible in utero (IU), around the time of birth [intrapartum (IP)], or postnatally (PN) through breastfeeding. Morbidity and mortality are associated with timing of HIV infection, with infants infected IU or IP having poorer outcomes than those infected PN.^4–6^ Infants who are exposed to maternal HIV but remain uninfected [HIV-exposed uninfected (HEU) infants] are also at risk of poorer health outcomes than infants not exposed to HIV.^7^

HIV-infected infants are at increased risk of low birth weight (LBW) and postnatal growth failure compared with HIV-uninfected infants.^8^ The effect of HIV exposure on growth remains uncertain, with heterogeneous results reported across sub-Saharan Africa.^7^ The Zimbabwe Vitamin A for Mothers and Babies (ZVITAMBO) trial, which was conducted in Zimbabwe before the availability of ART,^9^ provides an opportunity to evaluate the growth of infants born to HIV-infected mothers without the confounding effects of ART exposure. We describe linear and ponderal growth of children born to HIV-infected, compared with HIV-uninfected, mothers in Zimbabwe and explore the factors associated with growth failure among HIV-infected (IU, IP and PN) and uninfected (HEU and unexposed) children.

## MATERIALS AND METHODS

### Study Design

The ZVITAMBO trial of peripartum maternal and neonatal vitamin A supplementation was conducted between 1997 and 2001, as reported elsewhere.^4,6,9–12^ Briefly, 14,110 mother–infant pairs were enrolled within 96 hours of delivery from clinics in Harare, Zimbabwe. Mother–infant pairs were eligible if the infant was a singleton with birth weight ≥1500 g, the mother planned to stay in Harare after delivery, and neither mother nor infant had an acutely life-threatening condition. Medical care and counseling were offered throughout the trial.^13^ The trial preceded availability of ART for PMTCT or treatment in Zimbabwe, or recommendations for co-trimoxazole prophylaxis for HIV-exposed infants.

### Laboratory Testing and Ascertainment of HIV Status

Mothers were tested for HIV by enzyme-linked immunosorbent assay and western blot using previously described methods.^9^ Mothers testing HIV positive at baseline were retested to confirm infection; those who were HIV negative were retested at subsequent blood draws to detect seroconversion. CD4 counts were measured for HIV-positive mothers and a random selection of HIV-negative mothers at baseline using FACSCount (Becton Dickinson, Franklin Lakes, NJ). Hemoglobin was measured at baseline using a hemoglobinometer (HemoCue, Mission Viejo, CA) in all mothers enrolled from October 1998. For all children born to HIV-positive mothers, cell pellets and plasma were prepared from whole blood collected at baseline and all follow-up visits and stored at −70°C. At the end of the follow-up period, the last available sample from each child was tested for HIV by GeneScreen (Bernardsville, NJ) enzyme-linked immunosorbent assay on plasma if ≥18 months of age or by DNA polymerase chain reaction (PCR) assay Roche Amplicor version 1.5 (Roche Diagnostic Systems, Alameda, CA) in cell pellets if <18 months of age. If the last available sample was negative for HIV, the child was classified as HIV negative; if the last available sample was positive, earlier samples were tested to ascertain timing of infection. Children were classified into one of the 5 HIV-exposure categories: (1) unexposed: mother tested HIV negative at baseline; (2) HEU: mother HIV positive at baseline, infant HIV negative; (3) IU infected: infant PCR positive at baseline; (4) IP infected: infant PCR negative at baseline and PCR positive at 6 weeks of age and (5) PN infected: infant PCR negative at 6 weeks and HIV positive at a subsequent time point. Infants in the IP and PN groups contributed data to the HEU group before HIV acquisition. Infants were censored at their last HIV test result to avoid potentially misclassifying infants who had become infected before death or before the end of the study.

### Anthropometric Data

At baseline, infant birth weight was measured using an electronic scale (Seca model 727; Hanover, MD); infant birth length and maternal mid-upper arm circumference (MUAC) were measured according to methods described by Gibson.^14^ Gestational age was calculated using the Capurro method.^15^ Length and weight were measured at follow-up visits conducted at 6 weeks, 3 months and every 3 months to 12–24 months. Length and weight measurements for each child were converted to length-for-age (LAZ) and weight-for-length Z scores (WLZ) using World Health Organization international standards.^15^ Based on detailed feeding questionnaires, infants were classified into one of the 4 groups: nonbreastfed, exclusively breastfed (breast milk only plus vitamins and oral medications), predominantly breastfed (breast milk and nonmilk liquids) and mixed breastfed (breast milk plus nonhuman milks and/or solid foods), as previously described.^10^ This classification was limited to the first 3 months of life, because by 6 months, 93% of infants were mixed breastfed.^10^

### Statistical Analysis

The growth patterns of children from birth to 24 months were described by plotting mean LAZ and WLZ against age in months for each HIV infection/exposure group. Univariate logistic regression models were used to evaluate odds ratios (ORs) for stunting and wasting at baseline, 6 weeks, and 6, 12, 18 and 24 months, with HIV-unexposed children as the reference group.

For HIV-exposed and PN-infected children, time-varying exposure categories were used at each time point (ie, for each time point before becoming IP or PN infected, the child was classified as HEU). PN infants were only defined as PN infected at the first positive HIV test. As described above, infants were censored from analyses at the last HIV test result.

To identify factors associated with growth failure, a multivariable generalized estimating equation linear regression model for LAZ was constructed for each HIV infection/exposure group using an exchangeable correlation structure, taking into account within-child correlation between repeated measurements to create growth profiles between 6 weeks and 24 months. For the purposes of these models, infants were categorized based on infection/exposure status at 12 months of age (time-varying status not used). The covariates included in the models were independent predictors in univariate analyses that were not directly related to the definition of the child growth outcomes: infant sex, gestational age and feeding pattern at 3 months of age; maternal CD4 count, hemoglobin, parity (categorized into 3 groups: 1, 2–4 and ≥5), MUAC, death, marital status and education; household income; and vitamin A treatment arm. Only variables that were significant at *P* < 0.05 were retained in the models for each HIV-exposure group. All statistical analyses were conducted using STATA version 11.1 (StataCorp, College Station, TX).

### Ethical Approvals

Mothers provided written informed consent to take part in the trial, as approved by the Medical Research Council of Zimbabwe, Medicines Control Authority of Zimbabwe, Johns Hopkins Bloomberg School of Public Health Committee on Human Research and Montreal General Hospital Ethics Committee.

## RESULTS

At baseline, 4495 mothers were HIV positive and 9561 were HIV negative. Fifty-one had indeterminate results, and 3 were not tested; infants of these mothers were excluded. Of the 9561 mothers who were HIV negative at baseline, 351 seroconverted during follow-up; infants of these mothers were excluded. Thirty-three infants born to HIV-positive mothers were never PCR tested; these infants were excluded. Taken together, 438 (3.1%) of the 14,110 ZVITAMBO infants were removed from this current analysis, leaving 13,672 infants who were classified based on maternal HIV status and final infant HIV status as IU, IP, PN, HEU and unexposed. The number of participants in each group varied throughout the study period because (1) a proportion of HEU infants became HIV infected (IP or PN); (2) infants that acquired HIV with uncertain timing of infection were included in the analysis only at time points where their HIV status was certain; (3) study visit completeness was not 100%; (4) loss to follow-up and infant deaths occurred and (5) infants with incomplete results were censored at their final HIV test. Number of participants (N) included at each time point are shown in tables and figure legends.

### Baseline Characteristics

Table 1 describes baseline characteristics of mothers and infants, categorized by infection/exposure status at the end of the study (time-varying categories not used).

**TABLE 1.**
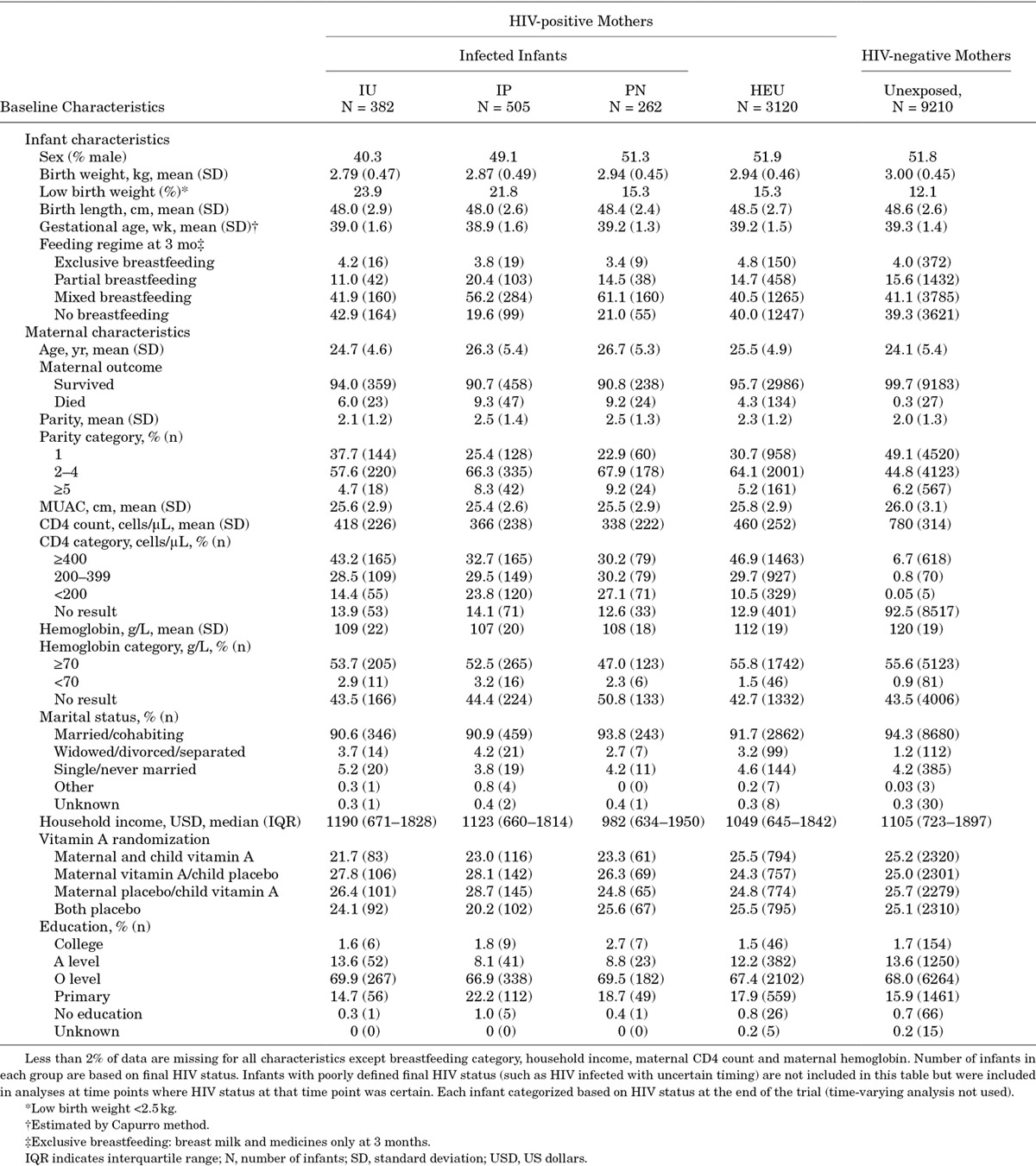
Baseline Characteristics by Maternal and Infant HIV Status and Timing of Infection

### Anthropometry at Birth

At birth, HIV-infected and HEU infants had significantly lower LAZ and WLZ and had more stunting and wasting than HIV-unexposed infants (Tables 2 and 3). Compared with unexposed infants, IU infants had twice the proportion of LBW (23.9% vs. 12.1%).

**TABLE 2.**
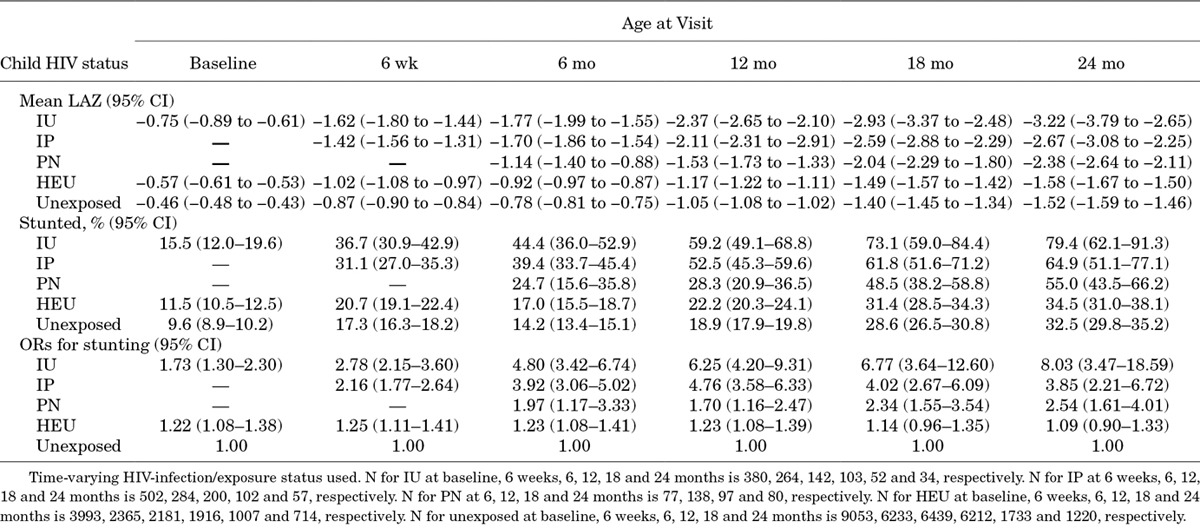
Mean Length-for-age Z Scores and Odds Ratios for Stunting According to Child HIV Status and Timing of Infection

### Postnatal Linear Growth

**FIGURE 1.**
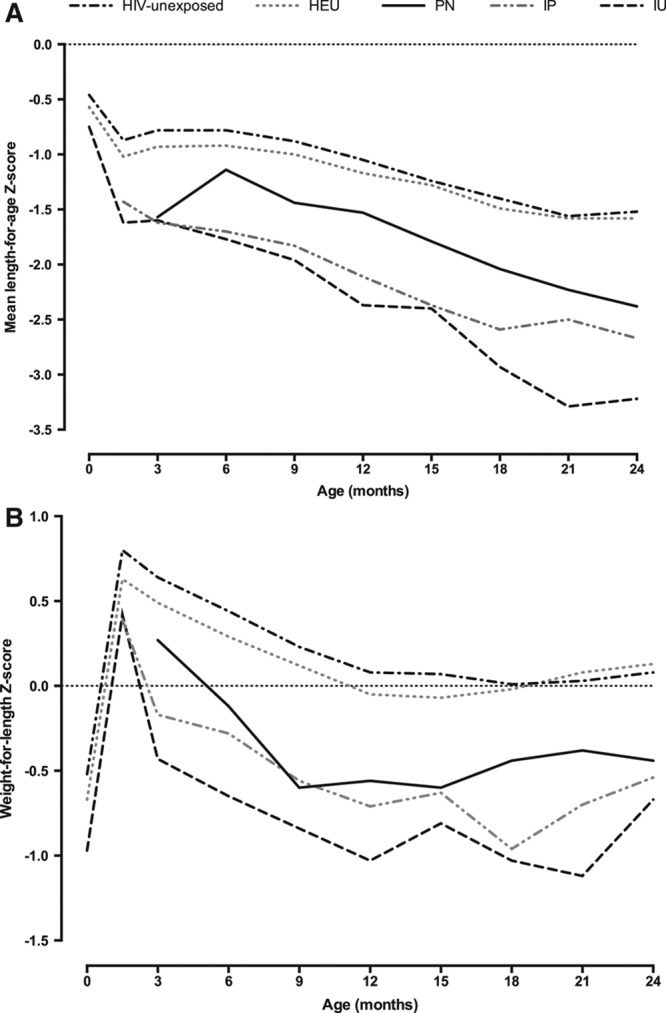
Mean length-for-age (A) and weight-for-length (B) Z scores from birth to 24 months according to child HIV status and timing of infection. A: N for IU at baseline, 6 weeks, 6, 12, 18 and 24 months is 380, 264, 142, 103, 52 and 34, respectively. N for IP at 6 weeks, 6, 12, 18 and 24 months is 502, 284, 200, 102 and 57, respectively. N for PN at 6, 12, 18 and 24 months is 77, 138, 97 and 80, respectively. N for HEU at baseline, 6 weeks, 6, 12, 18 and 24 months is 3993, 2365, 2181, 1916, 1007 and 714, respectively. N for unexposed at baseline, 6 weeks, 6, 12, 18 and 24 months is 9053, 6233, 6439, 6212, 1733 and 1220, respectively. B: N for IU at baseline, 6 weeks, 6, 12, 18 and 24 months is 342, 262, 142, 103, 52 and 34, respectively. N for IP at 6 weeks, 6, 12, 18 and 24 months is 449, 498, 282, 199, 102 and 56, respectively. N for PN at 6, 12, 18 and 24 months is 77, 138, 96 and 79, respectively. N for HEU at baseline, 6 weeks, 6, 12, 18 and 24 months is 3730, 2346, 2177, 1915, 1006 and 710, respectively. N for unexposed at baseline, 6 weeks, 6, 12, 18 and 24 months is 8598, 6206, 6431, 6202, 1733 and 1216, respectively.

There was a substantial drop in LAZ between birth and 6 weeks in all groups and a steady decline thereafter to around 21 months of age, with the drop being most pronounced in HIV-infected infants (Fig. 1A). Earlier timing of HIV infection was associated with more marked linear growth failure, with IU and IP children having lower LAZs than PN children at all time points. Linear growth was poorer in HEU than in HIV-unexposed children, with lower mean LAZ at all time points between birth and 24 months of age (Fig. 1A).

Stunting (LAZ: < −2) was common among all children in the study, but was especially frequent among HIV-infected children (Table 2). HIV-infected children had higher odds of stunting at all time points through 24 months compared with HIV-unexposed infants (Table 2). By 2 years of age, IU infants had 8-fold increased odds of stunting [OR: 8.03, 95% confidence interval (CI): 3.47–18.59] and IP-infected infants had almost 4-fold increased odds of stunting (OR: 3.85, 95% CI: 2.21–6.72) compared with HIV-unexposed children. HEU infants had significantly higher odds of stunting compared with HIV-unexposed infants until 12 months; beyond infancy, there remained a nonsignificant trend toward higher odds of stunting among HEU children.

### Postnatal Ponderal Growth

Mean WLZs increased from birth to 6 weeks in all groups; thereafter, there was a steady fall in WLZ through 18 months of age, with the decline most pronounced in HIV-infected children (Fig. 1B). Earlier timing of HIV infection was associated with more marked ponderal growth failure, with IU and IP children having lower WLZs than PN-infected children at all time points. Ponderal growth was poorer in HEU than in HIV-unexposed children, with lower mean WLZ at all time points between birth and 12 months of age (Fig. 1B).

Wasting (WLZ: < −2) was uncommon among children born to HIV-uninfected mothers, but was more prevalent in HEU infants, and was especially frequent among HIV-infected children (Table 3). HIV-infected children had higher odds of wasting at all time points through 24 months compared with HIV-unexposed infants (Table 3). By 2 years of age, IU- and IP-infected infants had around 7-fold increased odds of wasting compared with HIV-unexposed children. HEU infants had a significantly higher odds of wasting at all time points between birth and 12 months compared with HIV-unexposed infants, but not beyond infancy.

**TABLE 3.**
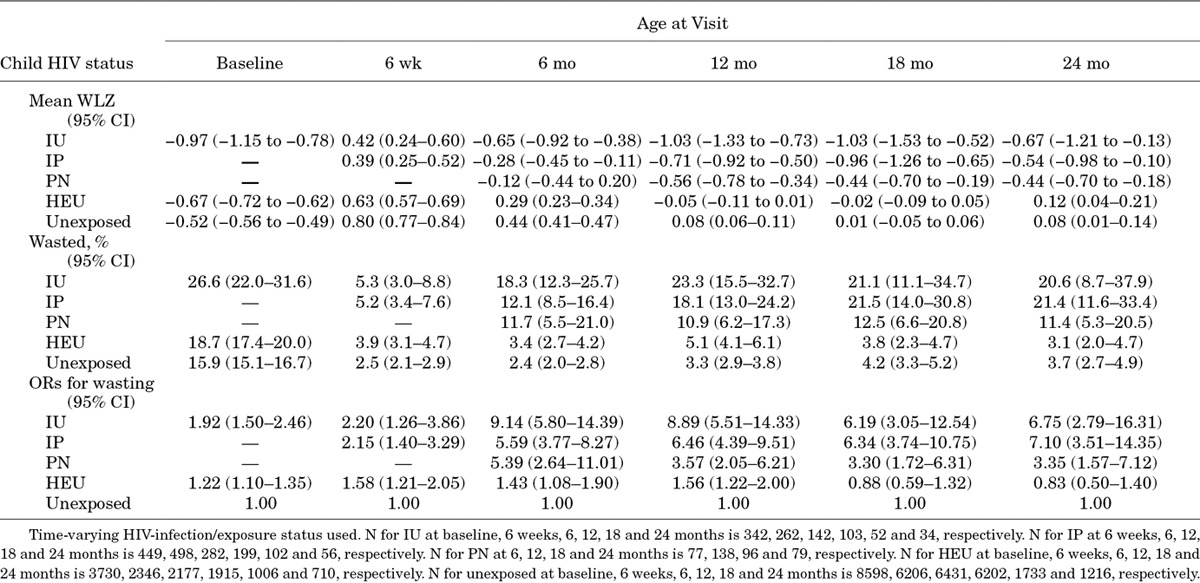
Mean Weight-for-length Z Scores and Odds Ratios for Wasting According to Child HIV Status and Timing of Infection

### Factors Associated With Growth Profiles According to Infection/Exposure Category

To understand the mechanisms underlying growth failure, we undertook generalized estimating equation models for each exposure category, identifying factors associated with linear growth profiles between 6 weeks and 24 months (Table 4). Gestational age was associated with linear growth in all groups. Male sex was associated with poorer growth in all groups except IU children. Maternal MUAC was associated with linear growth of HIV-uninfected, but not HIV-infected, infants. Breastfeeding status was associated with growth in IP and PN children. Maternal education was associated with linear growth in HIV-unexposed infants; though virtually all study mothers had at least primary education, HIV-uninfected mothers with more years of schooling had children with significantly greater LAZ in a dose-responsive pattern (college education, A level > O level > primary education, no education). Maternal marital status was associated with growth in PN and HEU infants; in HEU infants, there was a negative relationship between linear growth and the mother being widowed, divorced or separated, but all other social factors, including maternal death, parity, income and education and the mother being single, were not associated with linear growth of HEU infants.

**TABLE 4.**
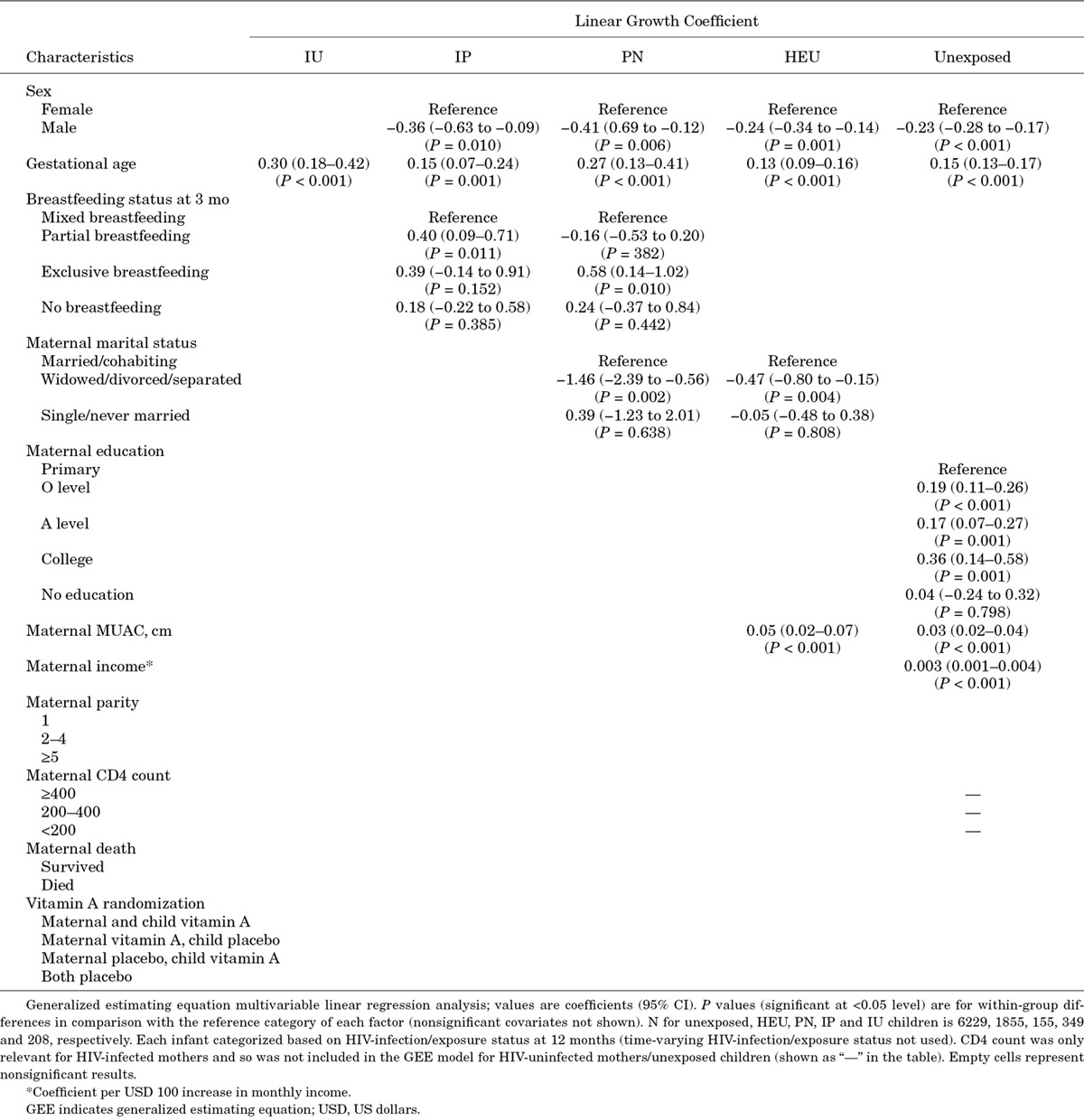
Generalized Estimating Equation Linear Regression Models for Mean Length-for-age Z Scores at 12 Months According to Maternal and Child HIV Status

## DISCUSSION

Zimbabwean infants followed from birth in the ZVITAMBO trial had poor growth compared with World Health Organization standards, highlighting the burden of undernutrition in this setting. HIV-infected children had the most profound growth failure, leading to a substantial burden of stunting and wasting in this population.

Regardless of HIV-exposure status, infants in this cohort had a sharp decline in LAZs from birth to 6 weeks, followed by a progressive decline to around 21 months. This pattern is typical of linear growth failure in developing countries: a pooled analysis of child growth patterns from 54 countries, using World Health Organization standards, showed LAZ fell dramatically from birth through 24 months, with little or no recovery thereafter.^16^ By contrast, WLZs increased over the first 6 weeks of life in all infants in our cohort, followed by a more gradual decline to around 18 months of age, leading to a substantial burden of wasting among HIV-infected children, but not among HIV-unexposed children.

The causes of stunting in developing countries are multifactorial.^17^ Although inadequate diet plays an important role, even the most successful nutrition programs improve linear growth only modestly.^18^ In a recent study of HIV-unexposed infants from this cohort, we found a strong relationship between recent illness and reduction in insulin-like growth factor-1 levels, highlighting acute infections as one important driver of growth failure.^19^ We also showed a relationship between inflammatory biomarkers during infancy and linear growth failure, which was mediated by reduced insulin-like growth factor-1, suggesting that stunting is a chronic inflammatory disease.^20^ Although the underlying causes of systemic inflammation are unclear, we and others have hypothesized that it is driven particularly by environmental enteric dysfunction, an almost ubiquitous pathology of the small intestine in conditions of poor hygiene and sanitation.^21^

The most striking finding in this study was the high burden of stunting and wasting in HIV-infected infants. Timing of HIV acquisition had an important impact on growth. IU- or IP-infected infants had earlier linear and ponderal growth failure than those infected PN through breastfeeding. This is similar to our previous finding from this cohort that mortality was related to timing of HIV infection.^4^ In the current study, the average LAZ fell below −2 by 6 months of age for IU-infected infants and by 9 months for IP-infected infants, compared with 18 months of age for those infected PN. A study of HIV-infected infants in India found similar results in the current PMTCT era; those with earlier HIV acquisition had poorer growth than infants infected later.^22^ The early onset of growth failure among HIV-infected infants demonstrates the rapidity with which untreated HIV affects growth, consistent with previous studies.^23–30^

A key finding from this study is that HEU infants had poorer growth than HIV-unexposed infants during infancy. Consistent with most^25,29,31–33^ but not all^34,35^ previous studies, HEU infants in this cohort had lower mean birth weights and a higher prevalence of LBW than HIV-unexposed infants, indicating an excess risk of IU growth failure; LBW and small-for-gestational-age in HEU infants are associated with increased mortality in the first year of life.^36–40^ By 6 weeks of age, LAZ and WLZ were significantly lower in HEU compared with HIV-unexposed infants and remained significantly lower throughout the first year of life. Compared with unexposed infants, HEU infants had 23% higher odds of stunting and 56% higher odds of wasting at 12 months. Comparisons of postnatal growth between HEU and unexposed infants from other settings have shown heterogeneous findings.^7^ Similar to the results of this analysis, a recent study from ART era, Botswana, of mostly formula-fed HEU infants and mostly breastfed HIV-unexposed infants (94.7% and 21.7% exclusively formula fed, respectively), found a 1.85-fold increased relative risk (RR) of stunting among HEU infants in the first year of life (95% CI: 1.03–3.31). Conversely, HIV-unexposed children had more stunting than HEU children between 1 and 2 years of age, which coincided with the weaning of HIV-unexposed children (RR: 1.56, 95% CI: 1.05–2.32); after 2 years of age, HEU children were again at increased risk of stunting (RR: 1.41, 95% CI: 1.06–1.88).^41^ Children were not followed past 2 years of age in the ZVITAMBO trial, but notably, breastfeeding status was not associated with linear growth of HEU infants.

The mechanisms underlying growth faltering among HEU infants in this cohort are unclear. Although it could be argued that stunting in HEU infants is more likely related to adverse social conditions rather than to HIV-related biologic mechanisms, maternal death, income, education and parity were not related to linear growth of HEU infants in this study. The relative contributions of factors associated with HEU infant growth remain uncertain. As mean LAZ in HEU infants was already lower at birth, the fetal environment may have a key influence on infant growth; HEU fetal growth restriction, and therefore infant growth restriction, may be at least partly driven by maternal HIV infection and inflammation during pregnancy.^42^ It is important to note that this study was conducted in the pre-ART era and infants were therefore exposed to untreated maternal HIV. However, we found no association between maternal disease severity (measured as CD4 count) and HEU infant growth, in contrast to mortality outcomes for this cohort.^4^ In Kenyan HEU children studied before ART availability, there was a 30% increased risk of stunting per log increase in maternal viral load,^43^ and in South African HIV-exposed infants, stunting, underweight and wasting were 1.7-fold, 1.6-fold and 1.6-fold higher, respectively, in infants born to mothers with CD4 <200 cells/μL compared with mothers with higher CD4 counts, suggesting that severity of maternal HIV can influence child growth.

This study has several strengths and limitations. Timing of infant HIV infection was carefully characterized, allowing us to evaluate differences in growth between IU, IP and PN groups. This is the largest cohort of HEU children to date, with a suitable comparison group of HIV-unexposed children. We censored infants at the last available HIV test, to ensure that the HEU group did not include undiagnosed PN children. However, very LBW infants (<1500 g) were excluded from the trial; because LBW was associated with postnatal growth, many infants excluded on this basis may have had poor postnatal growth. We used only WLZ to assess wasting in children, not MUAC; this could have influenced the proportion of children categorized as wasted. The high mortality among children may have led to survivor bias, especially among those with early HIV acquisition.

In summary, we show that growth failure is highly prevalent in Zimbabwean infants. In the absence of ART, HIV-infected infants had strikingly high rates of stunting and wasting, and earlier HIV infection was associated with poorer growth. Infants born to HIV-infected mothers, even in the absence of vertical HIV transmission, had poorer growth throughout the first year of life compared with those born to HIV-uninfected mothers. This study provides a strong rationale for strengthening efforts to eliminate mother-to-child HIV transmission and improving access to early infant diagnosis and co-trimoxazole prophylaxis, with ART initiation among those who become HIV-infected. These data suggest that early interventions are critical to tackle growth failure in sub-Saharan Africa.
